# Quality of life and research benefit and burden in siblings of children with life-limiting conditions: a prospective multicentre cohort study

**DOI:** 10.1186/s41687-026-01096-z

**Published:** 2026-05-23

**Authors:** Anne-Kathrin Gerber, Karin Zimmermann, Michèle Widler, Michael Simon, Nicolas von der Weid, Stefan Mitterer, Eva Bergsträsser

**Affiliations:** 1 Institute of Nursing Science, Department of Public Health, University of Basel, Bernoullistrasse 28, Basel, 4056 Switzerland; 2 Paediatric Palliative Care and Children’s Research Center, University Children’s Hospital Zürich, Lenggstrasse 30, Zürich, 8008 Switzerland; 3 Paediatric Palliative Care, University Children’s Hospital beider Basel (UKBB), Spitalsstrasse 33, Basel, 4056 Switzerland; 4 Division of Haematology-Oncology, University Children’s Hospital beider Basel (UKBB), Spitalsstrasse 33, Basel, 4056 Switzerland

**Keywords:** Siblings, Quality of life, Research burden, Palliative care, Pediatrics

## Abstract

**Background:**

Siblings of children suffering from a life-limiting condition (LLC) may experience impaired psychological and social well-being and strained family functioning. Due in part to concerns about research burden, evidence on siblings’ quality of life (QoL) remains limited. This study aimed to examine longitudinal changes in QoL among well siblings of children with a (LLC) during the palliative care and bereavement phases, and to assess their perceived benefits and burdens of research participation.

**Methodology:**

This prospective multicentre cohort study was conducted within the SPhAERA project. Data were collected using paper-and-pencil questionnaires at predefined care and bereavement time points. QoL was assessed using the KIDSCREEN-52 instrument, and perceived research benefit and burden were measured using eight self-developed items. Analyses were primarily descriptive. Siblings aged 8–18 years were recruited from three Swiss University Children’s hospitals between November 2019 and May 2022.

**Results:**

Twelve siblings participated. QoL scores were above the Swiss normative values across all domains except psychological well-being. QoL varied over time in all participants. The median research burden score was 1.4 (scale from 0 to 10), and no consistent benefits or burdens were reported.

**Conclusions:**

Care for children with a (LLC) should include screening to identify siblings with low QoL and increased support needs. Further research is required to examine factors influencing QoL and its fluctuations across the palliative and bereavement phases, in order to inform targeted support strategies.

**Supplementary Information:**

The online version contains supplementary material available at 10.1186/s41687-026-01096-z.

## Background

Living with, caring for, and mourning a child with a life-limiting condition (LLC) often impacts the psychological and social well-being of well siblings, who may be overwhelmed by changing care processes, family dynamics, parent relationships, caregiving responsibilities [1–6]. Consistent with family systems and family stress models, serious illness in one child can influence the functioning and well-being of the entire family, including siblings [7]. Compared with age-related norms, these siblings show significantly lower emotional, social, and school functioning [5, 6].

Based on parent- and self-reported data, overall psychosocial quality of life (QoL) ratings of siblings of children with a (LLC) commonly fall below their corresponding population norms [8]. Siblings of children receiving paediatric palliative care report not only significantly lower overall QoL, but also particularly low physical and psychological well-being and self-esteem compared with norms [9].

Additional burdens reported in the literature include increased internalising problems (e.g., anxiety and depression), and externalising problems (e.g., behavioural, social and diminished positive self-attributes) [1]. Moreover, bereaved siblings show increased anxiety, depression and illicit substance use [10–13]. While some siblings are able to adapt and cope with the burdens surrounding their sibling’s condition and death, others experience decreased QoL and psychosocial well-being [2, 6, 14–16].

However, current evidence on siblings of children with a (LLC) remains limited. First, most studies are cross-sectional and focus either on the palliative phase or on long-term outcomes after the child’s death. Second, available evidence relies largely on parental reports, which may differ considerably from siblings’ self-reports [17]. Researchers and clinicians, concerned about potentially overburdening well siblings often exclude them from active study participation [18].

Despite these concerns, evidence from palliative care research suggests that family members frequently perceive research participation as beneficial. However, only two studies have specifically examined the potential benefits and burdens of research participation among siblings [19]. In these studies, siblings reported that participation was certainly an emotional experience; nevertheless, the large majority considered it valuable, and none anticipated any long-term negative effects [20, 21].

This lack of self-reported evidence on QoL and perceived research burden limits the development of evidence-based clinical interventions to support siblings living with or bereaved by a sister or brother suffering from a (LLC). Longitudinal self-reported QoL data are needed to optimise support for siblings and ensure that their perspectives are represented. Data on siblings’ perceptions of the benefits and burdens of research participation are important to safeguard against potential overburdening.

## Methods

### Aims, design and setting

This prospective cohort study aimed (1) to examine the longitudinal development of QoL in well siblings of children with a (LLC), relative to Swiss and European norms, across the palliative care and bereavement phases, and (2) to report cross-sectionally on the well siblings’ perceived benefits and burdens of participating in a research study. The study is embedded within the SPhAERA project (Specialised Paediatric PAlliativE CaRe: Assessing family, healthcare professionals and health system outcomes in a multi-site context of various care settings). The SPhAERA project aimed to evaluate the effectiveness of a specialised paediatric palliative care programme by assessing clinical, service, and economic outcomes using a quasi-experimental comparative effectiveness approach [22]. Participating children with a (LLC) and their families received paediatric palliative care to varying degrees across different care settings, including hospitals, home care and long-term institutions. Further information regarding the SPhAERA study is available elsewhere [22]. Owing to the small sample of participating siblings, this cohort study is limited to describing the longitudinal development of QoL across the palliative and bereavement phases.

### Participants and recruitment

This study’s target population comprised siblings of children with a (LLC), e.g., congenital neurodegenerative conditions such as infantile neuroaxonal dystrophy, developmental and epileptic encephalopathies, and sequelae of traumatic brain injuries, who were recruited for the SPhAERA study. The SPhAERA study was conducted between November 2019 and May 2023 at three University Children’s hospitals in the German-speaking region of Switzerland and recruited patients with a (LLC), who might potentially require specialised paediatric palliative care, along with their families. Siblings were eligible to participate only if at least one parent both consented and participated in the study.

Siblings aged 8–18 years were eligible if they had sufficient proficiency in written German and were able to complete a questionnaire independently [22]. After receiving verbal and written information about the study, those who wished to participate provided either verbal assent (< 13 years) or written consent (≥ 13 years). Patients were recruited between November 2019 and May 2022 by care team members and local study coordinators.

### Data collection and management

Data were collected prospectively using paper-and-pencil questionnaires at predefined care time points (CT) and bereavement time points (BT). Following baseline assessment at study entry (CT0), questionnaires were sent out after 15 (CT1), 30 (CT2), 60 (CT3), 90 (CT4), 120 (CT5), 150 (CT6), 240 (CT7), and 330 days (CT8). If participants’ ill siblings died during data collection, bereavement data were collected at 30 (BT1), 120 (BT2), 210 (BT3), and 300 days (BT4) after the date of death.

The questionnaires were mailed to each participating family. If a questionnaire was not returned within one week of receipt, participants were reminded by telephone. At the regular study endpoint, either CT8 or BT4, the research burden questionnaire was included with the final questionnaire.

Information regarding the siblings’ context, including concomitant data about the ill child and the parents, was collected as part of the SPhAERA study through chart analysis and parental questionnaires. Participant pseudonymisation and data entry were performed in the secuTrial^®^ data management system according to the study manual [23].

### Measures

#### Quality of life

Siblings’ health-related QoL (HRQoL) was assessed using the German-language version of the KIDSCREEN-52 self-report measure. The KIDSCREEN-52 is a valid, sensitive, and conceptually and linguistically appropriate questionnaire used to assess the subjective health and the psychosocial, mental, and social well-being of children and adolescents in European and national health surveys [24]. The instrument comprises 10 domains: Physical Well-Being, Psychological Well-Being, Moods and Emotions, Self-Perception, Autonomy, Parent Relation and Home Life, Financial Resources, Peers and Social Support, School Environment, and Social Acceptance/Bullying. Its 52 items are scored on 5-point Likert scales assessing either the frequency of behaviours or feelings (never/seldom/sometimes/often/always), or the intensity of attitudes (not at all/slightly/moderately/very/extremely) [25]. Cronbach’s α coefficients for the 10 domains ranged from 0.77 (Social Acceptance/Bullying) to 0.89 (Financial Resources and Psychological Well-Being) [25].

Cronbach’s α coefficients were calculated for our study sample at study entry (CT0); however, given the small sample size, these estimates should be interpreted with caution. Cronbach’s α coefficients ranged from 0.56 (Financial Resources) to 0.90 (Moods and Emotions).

Although the KIDSCREEN-52 specifically measures HRQoL, in the context of the present study involving well siblings of children with a life-limiting condition, HRQoL is interpreted as an indicator of the broader construct of QoL. This interpretation is consistent with the definition of QoL proposed by the WHO as “*an individual’s perception of their position in life in the context of the culture and value systems in which they live and in relation to their goals*,* expectations*,* standards and concerns.*” [26].

Because the KIDSCREEN-52 does not provide a global HRQoL score, a general index was derived from the KIDSCREEN-52 items using the KIDSCREEN-10-Index. The KIDSCREEN-10 is a precise and stable HRQoL index with good internal consistency (Cronbach’s α = 0.82) and test-retest reliability (intraclass correlation coefficient = 0.70) [27, 28]. In our sample, Cronbach’s α coefficient at study entry (CT0) was 0.83.

KIDSCREEN data collected between 2001 and 2004 from 22,827 children and adolescents across 13 European countries provide normative KIDSCREEN scores [28]. European and national normative data are available at http://www.kidscreen.org. The KIDSCREEN-52 domain scores and KIDSCREEN-10 scores were transformed into a 0–100 scale according to the KIDSCREEN norm scale sum table with higher values indicating higher QoL.

#### Research benefit and burden

Inspired by Reggio et al., we assessed the perceived benefits and burdens of research participation using eight self-developed items (Table 1) [29].

**Table 1:** Self-developed items assessing the benefits and burdens of research participation

Item	Scale type	Response options and coding
Did you find participating in this study valuable?	4-point scale	1 = No, definitely not 2 = Rather no 3 = Rather yes 4 = Yes, definitely
Did participation in the study have negative effects for you?		
Did participation in the study have positive effects for you?		
If participation had negative and/or positive effects for you, what were they?	Open-ended	Free text
How did you find the length of the questionnaires?	4-point scale	1 = too minimal 2, 3 = adequate 4 = too extensive
How did you find the time required to participate in the study?		1 = adequate 2, 3^a^ 4 = too elaborate
Do you have additional comments about your participation in the study?	Open-ended	Free text
May we ask how burdensome you found your participation in the study?	Vertical visual analogue scale, 10 cm	10 = maximally burdensome 0 = not burdensome at all

^a^midpoints without semantic title of 4-point scale

#### Sociodemographic characteristics

Well siblings’ sociodemographic variables included gender, KIDSCREEN age category (8–11 years, 12–18 years), nationality, religion, school/education level, number of siblings, age relative to the ill sibling (older/younger), and family structure.

Ill children’s sociodemographic and condition-related variables included age (years), primary residence, diagnostic group (neurology/cardiology/oncology/other), illness duration (years from date of diagnosis to date of study entry) and phase of illness at study entry (stable/unstable). Illness phase was categorised by the study team using an adapted version of the Phase of Illness Tool in Pediatric Palliative Care [30].

Parental sociodemographic variables included household income in Swiss francs (CHF), education level.

### Data analysis

Sociodemographic variables were analysed using descriptive statistics (central tendency and dispersion measures, as well as percentages and frequencies). The general health-related QoL index during the ill child’s palliative and bereavement phase was visualised at the individual sibling level. Median health-related QoL domain scores derived from the KIDSCREEN-52 and assessed longitudinally during the palliative and bereavement phases were analysed using descriptive statistics and compared with Swiss and European self-reported population normative values retrieved from https://www.kidscreen.org/.

Fisher’s exact tests and odds ratios were used to assess differences in age group and sex distribution between our study sample and the normative samples. The Mann-Whitney U test was used to evaluate differences between participating and non-participating siblings. Research benefits and burdens were analysed using descriptive statistics.

Result tables and data visualisation were generated using the R (version 4.0.3.42) and the R packages *tableone* and *ggplot2* (version 3.4.4) packages. *p* -Values of < 0.05 were considered statistically significant [31].

#### Missing data

Missing data were analysed with regard to distribution and randomness. For the KIDSCREEN-52, 0.9% (*n* = 47) of responses were missing. Of these, 39 responses were classified as missing not at random. Examples included omitted items in the KIDSCREEN-52 domains *School Environment* or *Financial Resources*, for instance due to school holidays or because participants did not receive pocket money. Given the low number of responses missing at random, score calculation was restricted to complete cases.

## Results

Of 69 recruited families with 70 ill children, 26 siblings were eligible age-wise. Twelve siblings (46%) from 11 families consented to participate and completed data collection according to the study protocol. The participating siblings came from all three study centres, six of them from the one University Children’s Hospital which served as intervention site in the SPhAERA main study [22]. However, there is no data showing whether those siblings were supported by the specialised paediatric palliative care programme under study investigation. The median age of non-participating siblings (median = 12; interquartile range = 9.0–15.0) did not differ significantly from that of participants (median = 10.0; interquartile range = 8.5–13.0) (*p* = 0.35, *r* = 0.18). Five participants’ siblings died during the study (after CT0, CT1, CT5, CT6 and CT7 respectively). One sibling was seven years old at the time of study entry. Due to hers/his proficiency to understand written German and ability to independently complete the questionnaire, the study team decided to let that sibling participate.

Baseline characteristics are displayed in Table 2. Eleven (92%) participating siblings were Swiss, one (8%) German. All belonged to nuclear families. Ten (91%) ill siblings resided primarily at home, one (9%) in a long-term care institution. All participating fathers were employed full-time. Of the eleven participating mothers, eight (73%) were employed. The mothers’ mean full-time equivalency (FTE) was 0.36 individual FTE units (range = 0.1–0.55).

**Table 2:** Well siblings’ sociodemographic (*n* = 12), ill sibling sociodemographic and condition-related (*n* = 11), and parent (mothers: *n* = 11; fathers: *n* = 7) sociodemographic characteristics (at CT0/study entry)

	Characteristic / Category	
**Well siblings’ sociodemographic characteristics**	**Gender, *n* (%)**	
	Female	11 (92)
	Male	1 (8)
	**Age (years)**, ***n (%)***	
	7–11	9 (75)
	12–18	3 (25)
	Median (IQR)	10.0 (8.5–13.0)
	**Religion**, ***n*** **(%)**	*n* = 11
	Christian	8 (73)
	Non-denominational	2 (18)
	Islam	1 (9)
	**School / Education Level**, ***n*** **(%)**	
	Primary School (1–3 grade)	4 (33)
	Middle School (4–6 grade)	5 (42)
	Secondary school (7–9 grade)	1 (8)
	High School / Apprenticeship	2 (17)
	**Number of Siblings** ^a^, ***n (%)***	
	1	7 (58)
	2	3 (25)
	4	2 (17)
	**Age Relative to Ill Sibling**, ***n (%)***	
	Older	9 (75)
	Younger	3 (25)
**Ill sibling sociodemographic and condition-related characteristics**	**Age (y)**, ***n (%)***	
	0–6 years	4 (36)
	7–11 years	2 (18)
	12–18 years	5 (46)
	Median (IQR)	8.6 (6.2–13.1)
	**Diagnostic Group**, ***n (%)***	
	Neurology	8 (73)
	Oncology	3 (27)
	**Illness Phase**, ***n (%)***	*n* = 11
	Stable	7 (64)
	Unstable	4 (36)
	**Illness Duration (y)**	
	Median (interquartile range) Range	3.2 (0.3–9.0) 0.04–15.45
**Family and parents sociodemographic characteristics**	**Family/Household income in CHF**^**b**^, **n (%)**	*n* = 10
	50’000–99’999	5 (50)
	100’000–149’999	2 (20)
	150’000–200’000	3 (30)
	**Education**,** Mother**, ***n (%)***	
	Primary/secondary education or high school	0 (0)
	Vocational training	6 (55)
	College of higher education	4 (36)
	University degree	1 (9)
	**Education**,** Father**, ***n (%)***	
	Primary/secondary education or high school	1 (14)
	Vocational training	2 (29)
	College of higher education	3 (43)
	University degree	1 (14)

^a^ Including the ill child

^b^ Annual gross income, the average for Swiss families with children was CHF 144,676 from 2015–2017 [42]

### QoL in siblings of children with life-limiting conditions

**Fig. 1:**
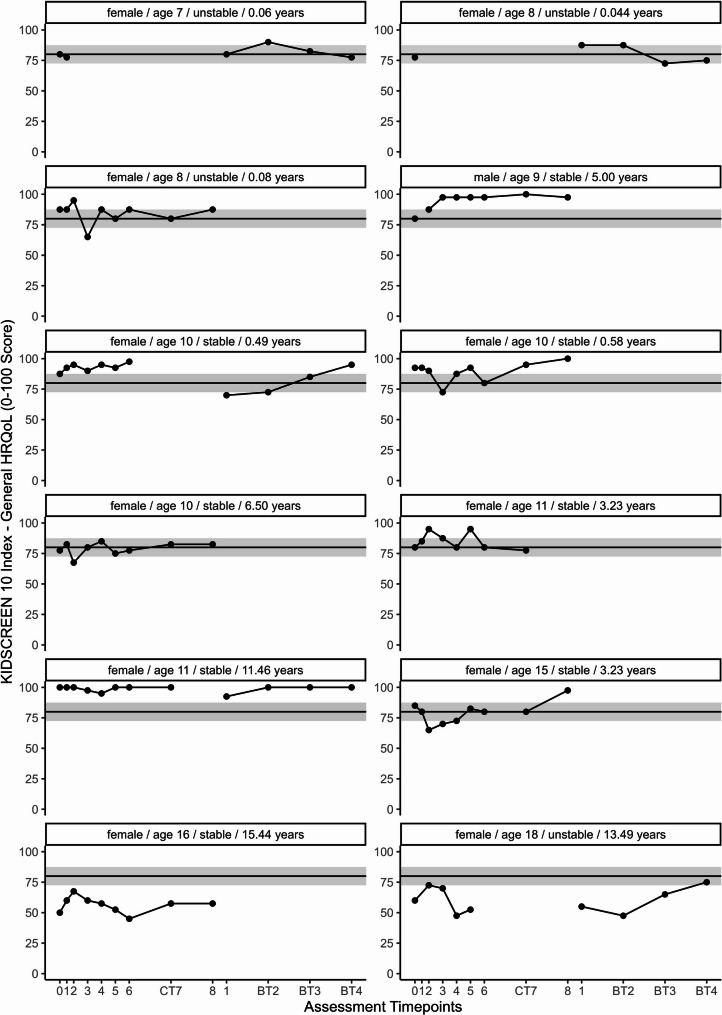
QoL trajectories of well siblings on an individual level in reference to the Swiss normative score. Well siblings’ QoL trajectories during their ill siblings’ palliative and bereavement phases are displayed. Each plot shows one well sibling’s QoL trajectory. The horizontal black line in each plot marks the Swiss norm population’s median (50th percentile); the shaded area above and below it covers the 25^th^–75^th^ percentiles. The plot title indicates the well sibling’s sex / well sibling’s age (years) / ill siblings’ illness phase / ill sibling’s illness duration. HRQL=Health-Related Quality of Life; CT=Care Timepoint (day 0=(CT)0, 15 days=(CT)1, 30 days=(CT)2, 60 days=(CT)3, 90 days=(CT)4, 120 days=(CT)5, 150 days=(CT)6, 240 days=CT7, and 330 days=(CT)8); BT=Bereavement Timepoint (30 days=(BT)1, 120 days=BT2, 210 days=BT3, and 300 days=BT4)

Figure 1 displays sibling’s general QoL index trajectory—individually and relative to the Swiss normative score—through the palliative and bereavement phase. Two siblings’ whose QoL scores were below the 25th norm percentile for almost their entire trajectories were female and belonged to the 12–18 years age group. Their ill siblings were younger and in the neurological diagnostic group.

Table 3 presents the median (50th percentile) and interquartile ranges (25th -75th percentile) of all ten KIDSCREEN-52 domains, including domain-level Swiss norm values. Additional file, Table 1 shows European norm population reference values. In the *psychological well-being* domain, our study sample median was below the Swiss norm, in all other domains it was above. There was a significant difference in age distribution (*p* = 0.005), with younger children appearing overrepresented in the study sample (OR = 5.80, 95% CI [1.63, 20.6]). Similarly, sex distribution differed significantly between groups (*p* = 0.008), with a higher proportion of females in the study sample (OR = 9.52, 95% CI [1.24, 72.9]).

**Table 3:**
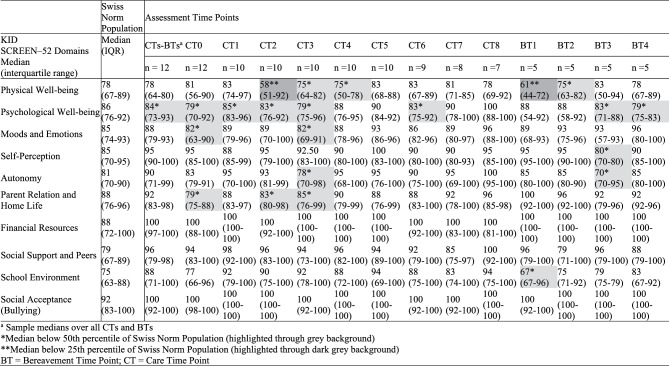
KIDSCREEN–52 domain scores: medians and interquartile ranges for all CT and BT timepoints

### Self-perceived benefit and burden of research participation in siblings of children with life-limiting conditions

Of the twelve participants, 10 (83%) completed the research burden questionnaire. The median perceived burden score was 1.4 (interquartile range = 0.9–3.25, range = 0.1–6.8). Table 4 reports perceived benefits and the positive and negative aspects of research participation.

**Table 4:** Benefit and burden of research participation in well siblings (*n* = 10)

Item		*n* (%)
Did you find participating in this study valuable?	yes, definitely	1 (10)
	rather yes	4 (40)
	rather no	5 (50)
	No, definitely not	0 (0)
Did participation in the study have negative effects for you?	no, definitely not	8 (80)
	rather no	1 (10)
	rather yes	1 (10)
	yes, definitely	0 (0)
Did participation in the study have positive effects for you?	yes, definitely	1 (10)
	rather yes	3 (30)
	rather no	6 (60)
	no, definitely not	0 (0)

Nine reported that the questionnaire length and time required for study participation were adequate. One considered the questionnaires too extensive (questionnaire length) and too elaborate (time requirement). Only one answered the open-ended question regarding positive/negative consequences: “None, except that I received a pen. And I may have thought about my life.”

## Discussion

This study examined the development of siblings’ QoL, relative to normative data, across their ill siblings’ palliative and bereavement phases, and assessed the perceived benefits and burdens of research participation. Our data suggest variability in participants’ QoL over time, although these observations should be interpreted cautiously given the descriptive nature of the analyses. Median QoL scores—across all CTs and BTs—were above the 50th percentile of European and Swiss norms, except for the *psychological well-being* domain, which was below the Swiss norm. Two participants repeatedly scored below the 25th percentile. Overall, siblings reported participation as neither particularly beneficial nor harmful, and reported a low burden.

Importantly, the use of siblings’ self-reported outcomes provides insights that may not be fully captured through parental proxy reports. Previous studies have demonstrated only moderate agreement between sibling self-reports and parent-proxy reports, with parents tending to underestimate internal experiences such as emotional functioning and well-being [32, 33]. Houtzager et al. further highlighted that discrepancies between informants are particularly pronounced in less observable domains, underscoring the importance of including siblings’ own perspectives [32]. In line with this, our findings of largely normative QoL alongside reduced psychological well-being illustrate a nuanced pattern that may not be detected through proxy reporting alone. Moreover, siblings’ self-reported low research burden contrasts with common assumptions about overburdening this population. Together, these findings support the inclusion of sibling self-report as a critical component in both research and clinical assessment.

Our findings align with those of Adler and Schraner, who reported largely normative QoL in 103 Swiss siblings aged 5-18-years of children and adolescents with disabilities or chronic conditions (KIDSCREEN-27), with the exception of lower psychological well-being [34]. In contrast, international studies suggest less favorable outcomes. Dinkelbach et al. reported lower self-reported total QoL, self-esteem, and physical and psychological well-being among German siblings of children receiving paediatric palliative home care, compared to the national norms measured with the KINDL instrument [9]. A meta-analysis identified a small overall negative effect in siblings of children with chronic health conditions, including increased internalising and externalising problems and fewer positive self-attributes [1]. Similarly, siblings of children with life-limiting conditions in California self-reported poorer emotional, social and school functioning compared to regional norms [5].

Time since diagnosis may be crucial in explaining these differences. In our sample, the ill siblings’ median illness duration was 3.2 years (range: 16 days to 15 years). Houtzager et al. assessed the association of coping and family functioning with psychosocial adjustment in siblings at 1, 6, 12 and 24 months after cancer diagnoses [15]. They found that QoL declined shortly after diagnosis, improved over the following year, and approximated reference levels after two years [15]. Together with our findings, this suggests substantial resilience and adaptability in siblings over time. Resilience has been positively associated with emotional, social, and school functioning [5], and siblings develop coping strategies such as acceptance of one’s situation, active management of fear, and communicating openly to family and friends [35]. The observed QoL fluctuations in our study likely reflect ongoing adaptation to changing circumstances.

Lower QoL in our two older participants may indicate greater vulnerability among older siblings. Previous studies have reported lower QoL in adolescents compared to younger siblings, as well as greater psychological difficulties among older siblings and females [9, 15]. This is consistent with our findings, where the lowest QoL scores were observed in the oldest participants, all of whom were female. These results underscore the importance of routine screening to identify siblings at risk and address their support needs, with particular attention to age [1].

Data collection coincided with the coronavirus disease 2019 pandemic, which may have influenced QoL outcomes. Ravens-Sieberer et al. reported an increase in the prevalence of low QoL (KIDSCREEN-10 Index) among German children and adolescents, rising from 15.3% pre-pandemic to 47.7% between December 2020 and January 2021. By September to October 2022, this proportion had declined to 27.0% [36]. Similarly, Swedish youth (11–19 years) reported lower QoL across all KIDSCREEN-27 domains during the pandemic compared to Swedish pre-pandemic national norms, with females consistently reporting lower scores across domains [37]. In our sample, the relatively high proportion of younger participants (7–11 years), who may have been less impacted by the pandemic, could have attenuated this effect. Additionally, siblings of children with a (LLC) may develop enhanced coping capacities and higher resilience, allowing them to navigate challenging situations more effectively.

Researchers’ and clinicians’ reluctance to involve family members for palliative care research often reflects concern about adding to their existing burdens [19, 38–40]. However, evidence indicates that burden is frequently overestimated and benefits underestimated [19]. For instance, in a study of bereaved siblings, 84% described participation as a positive experience, and none anticipated long-term negative effects [21]. Similarly, research participation has not been found to adversely affect siblings in families of children with a (LLC) [20]. Stevens et al. concluded that research involving families with a child suffering from a (LLC) can be conducted safely when appropriate preparation and support are provided [20]. Systematic assessment of participation-related benefits and burdens is recommended in the field [19]. Overall, available evidence indicates that research participation does not negatively affect siblings and their perspectives provide valuable insights distinct from those of their parents [9, 41].

### Strengths and weaknesses

As previous research in this field has predominantly relied on parental proxy reports, this study deliberately prioritised siblings’ self-reported outcomes to capture their perspectives directly. The longitudinal design enabled the examination of changes in siblings’ QoL over time and may help identify periods along the ill child’s disease trajectory that are particularly challenging for siblings, as well as potential effects of paediatric palliative care interventions or support programmes [2, 9]. A further strength is the use of a validated, multidimensional QoL instrument, allowing comparison with normative data. In addition, assessing well siblings’ self-reported research burden at the end of the palliative or bereavement phase represents a novel contribution.

However, the use of convenience sampling and the parental pre-selection of participants may have introduced selection bias. Non-participating siblings may have experienced higher levels of stress and burden, and lower QoL, than those who participated. The small sample size limits the generalisability of the findings and resulted in a sample that was predominantly female, with only one male participant. Furthermore, in the absence of a suitable validated instrument to assess research burden, a study-specific measure was developed, limiting comparability across studies. Apart from QoL, no variables were assessed longitudinally, and the small sample size restricted analyses to descriptive statistics, precluding the examination of factors influencing QoL fluctuations. Finally, although the potential impact of the coronavirus disease 2019 pandemic on siblings’ QoL was not directly assessed, some influence cannot be excluded.

## Conclusions

Care for children with a (LLC) should include careful consideration of how and when to offer proactive support for their siblings, particularly those with low QoL. Targeted screening is needed to identify siblings at risk of reduced QoL and increased burden. To inform tailored support strategies, future research should examine factors influencing QoL and its fluctuations across the palliative and bereavement phases. For particularly vulnerable siblings, further research is required to identify ways to strengthen resilience and coping. Importantly, research should not focus solely on deficits but should also capture the diversity of sibling outcomes.

Because existing evidence on siblings’ QoL and other outcomes relies largely on parental proxy reports, evidence on research burden remains sparse. Our findings therefore should encourage researchers and clinicians to include siblings’ self-reported perspectives in their studies. Further work is needed to optimise research participation for siblings by minimising burden and potential negative effects while enhancing positive or meaningful experiences. In addition, strategies to improve recruitment—particularly among older siblings—should be explored, for example by integrating research activities with existing support services or by tailoring approaches to adolescents’ needs. Future research may also benefit from combining sibling and parent reports to examine informant agreement.

## Electronic Supplementary Material

Below is the link to the electronic supplementary material.

Supplementary Material 1

## Data Availability

The datasets generated and/or analysed during the current study are not publicly available due to confidentiality reasons but are available from the corresponding author on reasonable request.

## Abbreviations

BT: Bereavement timepoint
CHF: Swiss Franc
CT: Care timepoint
FTE: Full-time equivalent
HRQL: Health-related quality of life
LLC: Life-limiting condition
QoL: Quality of life
SPhAERA: Specialised Paediatric PAlliativE CaRe: Assessing family, healthcare professionals and health system outcomes in a multi-site context of various care settings
WHO: World Health Organization
