# Supplementary material for: Quality of life and research benefit and burden in siblings of children with life-limiting conditions: a prospective multicentre cohort study
Source: J Patient Rep Outcomes. 2026 May 23;10:123. doi: 10.1186/s41687-026-01096-z (PMC13385311; doi:10.1186/s41687-026-01096-z)
Supplement: Supplementary file 1 — Supplementary Material 1 [file 41687_2026_1096_MOESM1_ESM.docx]

**Additional file:** Table 1 presents our sample’s median (50^th^ percentile) and interquartile ranges (25^th^–75^th^ percentile) for all ten KIDSCREEN–52 domains. Reference values of European norm population are provided on the per-domain level.

**Table 1:** KIDSCREEN-52 domain scores: medians and interquartile ranges for all care and bereavement assessment timepoints

|  | European Norm Population | Assessment Time Points | | | | | | | | | | | | | |
| --- | --- | --- | --- | --- | --- | --- | --- | --- | --- | --- | --- | --- | --- | --- | --- |
| KID  SCREEN–52 Domains  Median  (interquartile range) | Median  (IQR) | CTs-BTs^a^ | CT0 | CT1 | CT2 | CT3 | CT4 | CT5 | CT6 | CT7 | CT8 | BT1 | BT2 | BT3 | BT4 |
|  |  | n = 12 | n =12 | n =10 | n =10 | n =10 | n =10 | n =10 | n =9 | n =8 | n =7 | n =5 | n =5 | n =5 | n =5 |
| Physical Well-being | 72  (56–83) | 78  (64-80) | 81  (56-90) | 83  (74-97) | 58*  (51-92) | 75  (64-82) | 75  (50-78) | 83  (68-88) | 83  (67-89) | 81  (71-85) | 78  (69-92) | 61*  (44-72) | 75  (63-82) | 83  (50-94) | 78  (67-89) |
| Psychological Well-being | 79  (67–92) | 84  (73-93) | 79  (70-92) | 85  (83-96) | 83  (76-92) | 79  (75-96) | 88  (76-95) | 90  (84-92) | 83  (75-92) | 90  (78-100) | 100  (88-100) | 88  (54-92) | 88  (58-92) | 83  (71-88) | 79  (75-83) |
| Moods and Emotions | 82  (68–93) | 88  (79-93) | 82  (63-90) | 89  (79-96) | 89  (70-100) | 82  (69-91) | 88  (78-96) | 93  (86-96) | 86  (82-96) | 89  (80-97) | 96  (88-100) | 89  (68-93) | 93  (75-96) | 93  (57-93) | 96  (80-100) |
| Self-Perception | 75  (60–90) | 95  (90-100) | 95  (85-100) | 88  (85-99) | 95  (79-100) | 92.50  (83-100) | 90  (80-100) | 100  (83-100) | 90  (80-100) | 90  (80-93) | 95  (85-100) | 95  (95-100) | 95  (90-100) | 80  (70-80) | 90  (85-100) |
| Autonomy | 75  (60–90) | 90  (71-99) | 83  (79-91) | 95  (70-100) | 93  (81-99) | 78  (70-98) | 95  (68-100) | 95  (76-100) | 90  (75-100) | 95  (69-100) | 100  (95-100) | 85  (80-100) | 85  (80-90) | 70*  (70-95) | 85  (80-100) |
| Parent Relation and Home Life | 83  (67–96) | 92  (83-98) | 79*  (75-88) | 88  (83-97) | 83  (80-98) | 85  (76-99) | 90  (79-99) | 88  (76-99) | 88  (83-100) | 92  (78-100) | 96  (85-98) | 100  (92-100) | 96  (92-100) | 92  (79-96) | 92  (92-96) |
| Financial Resources | 75  (50–92) | 100  (97-100) | 100  (88-100) | 100  (100-100) | 100  (92-100) | 100  (100-100) | 100  (100-100) | 100  (100-100) | 100  (92-100) | 100  (83-100) | 100  (81-100) | 100  (100-100) | 100  (100-100) | 100  (100-100) | 100  (100-100) |
| Social Support and Peers | 75  (63–88) | 96  (79-98) | 94  (83-100) | 98  (92-100) | 96  (83-100) | 94  (73-100) | 96  (82-100) | 94  (89-100) | 92  (79-100) | 85  (75-97) | 100  (92-100) | 96  (79-100) | 79  (71-100) | 96  (79-100) | 88  (79-100) |
| School Environment | 67  (54–83) | 88  (71-100) | 77  (66-96) | 92  (79-100) | 90  (75-100) | 92  (78-100) | 88  (72-100) | 94  (69-100) | 88  (75-100) | 83  (74-100) | 94  (75-100) | 67  (67-96) | 75  (71-92) | 79  (75-79) | 83  (67-92) |
| Social Acceptance (Bullying) | 92  (83–100) | 100  (92-100) | 100  (98-100) | 100  (100-100) | 100  (100-100) | 100  (92-100) | 100  (100-100) | 100  (100-100) | 100  (92-100) | 100  (92-100) | 100  (100-100) | 100  (92-100) | 100  (100-100) | 100  (100-100) | 100  (100-100) |
| ^a^ Sample medians over all CTs and BTs  *Median below 50th percentile of Swiss Norm Population (highlighted through grey background)  BT = Bereavement Time Point; CT = Care Time Point | | | | | | | | | | | | | | | |
